# Do we urologists know enough about gender minorities with prostate cancer?

**DOI:** 10.1590/S1677-5538.IBJU.2023.9914

**Published:** 2024-03-18

**Authors:** Valter Javaroni

**Affiliations:** 1 Hospital Federal do Andaraí Rio de Janeiro Departamento de Andrologia Rio de Janeiro RJ Brasil Departamento de Andrologia, Hospital Federal do Andaraí Rio de Janeiro, Rio de Janeiro, RJ, Brasil

Medical training, particularly that required by specialists dealing with sexuality, takes time. It is not just a matter of accruing information, but mainly of acquiring practical experience. This is not to mention the development of maturity, with sufficient sensitivity to deal with the sexuality of others, always considering biopsychosocial aspects and respecting all individualities.

Those with more than 20 years of urological practice rarely had the opportunity to attend classes on gender identity and sexual orientation during their college training. On the other hand, they are at the height of their careers and have accumulated large knowledge and experience, at the apex of being able to help patients. Would this statement be true if the complaint is in the sexual area, including from a transgender woman?

This question arises because we have never experienced so many and such rapid transformations in human sexuality as in the last 20 years. Practitioners are faced with the paradox of having many years of training, broad theoretical knowledge, great practical experience in a specific area, with application of minimally invasive modern technology, while still being considered novices in the management of the health of a previously invisible group: the lesbian, gay, bisexual, transgender, queer, intersex, asexual, and all sexual and gender minorities (LGBT+).

How many urologic patients were regularly asked about sexual orientation in periodic appointments during the 2000-2010 period? How many urologists know the differences in anal sex and other sexual practices outside heteronormativity when dealing with cases of premature ejaculation, low sexual desire, or erectile dysfunction? How many good papers have you read on queer anorgasmia? How would you advise cancer screening or examine a transgender woman’s prostate? How often does the urologist explain the changes in the sex life of an asexual cisman with prostate cancer pending radical surgery? In short, are you comfortable in providing health care to transgender people?

This is one of the reasons to congratulate Drs. Dickstein and Collegues for their recent publication (1). This excellent paper helps to improve LGBT+’s health care not only by highlighting the lack of scientific information about this specific group, but mainly by listing some of the peculiarities that require adaptations and changes in relation to the type of assistance usually offered to the cisgender population concerning prostate cancer and sexuality.

Dr. Ross stated in 2006 after being elected president of the American Urological Association: "*Experience has taught the medical profession that action, change, and adaptation are the rule as novel technologies and therapies are introduced into the mainstream of medical care. Sexual medicine is no exception."* (2). Medical care also needs to identify, monitor, adapt to sociocultural changes. And the reality is that most of us (between 50 and 80 years old) were educated and trained in a heteronormative setting and the overwhelming majority of scientific medical evidence has been generated from clinical studies with cisgender and heterosexual individuals (3). Are we adopting a real patient-centered framework in our clinical practice (4)?

Individuals with disabilities along with lesbian, gay, bisexual, and transgender (LGBT) people, as well as racial and ethnic minority populations have differences in health care needs that result in health disparities (5). Despite undeniable advances and greater interest in understanding these differences and their impact on quality of assistance, progress is still slow and heterogeneous, having significant geographic variation (6). We have lost the opportunity to learn from the HIV epidemic. Not equating sexual behavior to sexual identity should become a basic rule. We also could have been able to increase our awareness of the health needs of LGBT+ persons (7).

The LGBT+ community has historically suffered discrimination, and has often been overlooked when discussing health care disparities. They continue to face barriers to equitable care. Stigma and discrimination, poverty, lack of education, racial or ethnic minority status, and other psychological health determinants keep LGBT+ people from accessing the care they need (8-10). Nearly one-fourth of transgender patients who participated in the 2015 U.S. Transgender Survey indicated that they did not seek medical attention for fear of being mistreated (11). Worldwide, transgender and gender diverse people commonly experience transphobia, stigmatization, ignorance, and even rejection when seeking health care services, which contributes to significant health disparities. Transgender people often report having to teach their medical providers how to care for them due to the latters’ insufficient knowledge and training (12). The recognition and understanding of this scenario should motivate healthcare providers to undertake efforts to guarantee the necessary welcoming attitude, ensure respect of social names, provide well-trained and qualified professionals able to understand the reality and biopsychosocial context of everyone (13). In other words, we need to provide qualified and customized health services for all.

Transgender healthcare is a rapidly evolving interdisciplinary field. In the last decade, there has been an unprecedented increase in the number and visibility of transgender and gender diverse people seeking support and gender-affirming medical treatment, in parallel with a significant rise in the scientific literature in this area. The World Professional Association for Transgender Health (WPATH) is an international multidisciplinary professional association whose mission is to promote evidence-based care, education, research and public policies, along with respect in transgender health, in its eighth iteration (12). Across successive iterations of the guidelines there is a trend both of reducing the stigma against transgender individuals and a shift in ethical considerations from "do no harm" to the core principle of patient autonomy. The requirement for universal mental health provider involvement, initially formulated via expert opinions, has not been retained in the most recent World Professional Association for Transgender Health Standards of Care. This has helped reduce barriers to care and connect more people who desire it to gender affirming care (14). Another advance came from the 11th edition of the International Statistical Classification of Diseases and Related Health Problems (ICD-11), which finally defined "depsychopathologized" gender incongruence to reflect evidence that transgender and gender diverse identities are not conditions of mental ill health (15). It is also true that research into LGBT+ health has been expanding as the community has become more visible and outspoken about engaging the healthcare system in developing a knowledge based on the distinctive challenges and health disparities they face (16).

Despite initiatives and undeniable progress, the recent COVID pandemic revealed a situation still far from comfortable. The LGBT+ communities have been affected the most by the 2019-Coronavirus disease (COVID-19) and the inequity in healthcare delivery and social security towards disadvantaged strata of society reemerged during this critical period like never before. We are still far from providing non-discriminatory, equitable and high-quality healthcare service regardless of the gender or sexual orientation of patients, and much more needs to be done to achieve equity for LGBT persons in the healthcare system. (17-19).

In Brazil, a country where on average more than one sexual gender minority person is murdered every day because of their sexuality or gender identity (the highest reported homicide rate in the world) (20), we face major challenges, including: access of the LGBTI+ population to the Brazilian Unified Health System (SUS); the need to train healthcare professionals; the decentralization of health services sensitive to the LGBTI+ population; the distinct forms of violence and discrimination; and the lack of research in health care conducted with specific groups, such as lesbians, bisexuals, intersex and other sexual minorities (21). A recent cross-sectional study was carried out in Brazil through a confidential online questionnaire with more than 6,500 participants (1,332 LGBT+ and 5,361 non-LGBT+) with a median age of 60 years and showed that being LGBT+ was an independent factor associated with worse access to health (PR = 2.5, 95% CI 2.04-3.06). The rate of screening for breast, colon, and cervical cancer was also found to be lower in the LGBT+ population (22).

Approximately 0.4-1.3% of the global population is transgender. Estimates for some countries are as high as 1.2% (23). As society at large begins to better recognize and understand the social and psychological issues surrounding transgender patients, more transgender individuals will feel comfortable in seeking urologic care, so urologists need to be better educated about social, behavioral, physiological, and anatomical issues that face transgender patients (24).

In the largest American national transgender survey to date (n=6,456), 30% of the respondents reported current smoking (1.5x the rate of the general population), 26% reported current or former alcohol or drug use to cope with mistreatment, and 41% reported having attempted suicide (26x higher than the general population) (25). Transgender women are internationally recognized as a population group that carries a disproportionate burden of HIV infection, with a worldwide HIV prevalence of 20% (26). The Center for Disease Control and Prevention reported that in 2013, 1.9% of HIV tests performed on transgender individuals were positive, compared to 0.9% for cisgender males and 0.2% for cisgender females (27). The estimated prevalence of HIV among transgender women of reproductive age (range 15–49) is 21.7% (95% CI: 18.4-25.1%), which is 34 times higher than for cisgender adults in the same age range (28). A US sample of 1,093 transgender persons demonstrated high prevalences of clinical depression (44.1%), anxiety (33.2%), and somatization (27.5%) (29).

In addition to the usual care, transgender patients often require medical interventions such as hormone therapy and/or surgery (30). In transgender women, gender identity can be expressed through any combination of name, pronoun, hairstyle, clothing, and social role. Feminization can also include several medical and surgical interventions. Some transgender individuals want to transition medically by taking gender-affirming hormones (GAH) and/or pursuing gender-affirming surgery (GAS). The main goal is to deprive the phenotypically masculine body of androgens and simultaneously provide estrogen therapy for feminization (14, 31).

Transgender aging is an underexplored field and there is little data available in the medical literature, despite the increasing life span and greater visibility of the transgender population (32). Over the last 50 years, cancer mortality has decreased. The leading contributor to this decrease has been the widespread adoption of cancer screening protocols, but in the case of transgender and gender-diverse people, evidence-based data is lacking (33). Among transgender women, the need for ongoing screening for prostate cancer is not well determined. Little is known about prostate cancer screening in this population since there are still many questions concerning this group, such as: understanding the risks/benefits of prostatic specific antigen (PSA) screening; determining how best to mitigate potential negative psychological effects of PSA screening; establishing baseline PSA values for those on GAH (and determining what values should be considered "elevated"); establishing when to initiate PSA screening for those on GAH; and establishing the accuracy of biomarkers for those undergoing GAH (34, 35).

LGBT+ people with cancer are at higher risk of distress and impaired quality of life compared with non-LGBT+ people, and one possible reason is the fact that they already deal with minority stress and lack of social support, which can mean greater difficulty in overcoming cancer diagnosis and impair wellbeing (36). Due to limitations of existing cohort studies, the true incidence of prostate cancer in transgender women is unknown, but is thought to be less than the incidence among cisgender males (31). Transgender women are extensively under-represented in national cancer databases, a fact that hinders the evidence for this growing population concerning prostate cancer epidemiology and the creation of professional guidelines (37) to base specific screening recommendations (38). While clear guidelines exist on the role of screening, diagnosis, management and outcomes in cis males, there is no evidence-based guidance for clinicians regarding transgender women (39). The World Professional Association of Transgender Health (WPATH) and the Endocrine Society advocate that transgender females should be offered the same screening program as a cisgender men based on the lack of strong evidence to suggest otherwise (12). It is important to consider that transgender patients’ cancer screening needs will vary by "what stage of their transition" they are in, since the start of GAH, non-genital and genital GAS, and surgical removal of some or all their reproductive organs may affect cancer risk (33). In a recent cohort evaluation including 2,957 transgender women, Premo H. et al. identified significantly lower PSA screening rates among transgender individuals for ages 40-54 and 55-69, but higher rates within the 70-80 age group (40).

Vaginoplasty is the most frequently performed gender-affirming genital surgery for gender-diverse people with genital gender incongruence. The procedure is performed to create an aesthetic and functional vulva and vaginal canal that enables receptive intercourse, erogenous clitoral sensation, and a downward-directed urine stream. In GAS for transgender females, the prostate is usually not removed (41). It should be emphasized that there is no "one-size-fits-all" approach, and transgender people may need to undergo all, some or none of these interventions to support their gender affirmation (12).

However, doctors should be aware of the influence of hormonal therapy and GAS on sexual functioning and satisfaction (16, 42). Some evidence now exists of the long-term impact of GAS on sexual wellbeing. But there are no data on sexual wellbeing following orchiectomy-only, vocal feminization surgery, facial feminization surgery or the removal of the female sexual organs. So, there is a need for more studies focusing exclusively on the effects of GAS on sexual wellbeing (43, 44). Current understanding of the effect of chronic disease on LGBT+ sexuality is limited and mostly focused on the male sexual response. LGBT+ persons who have trouble with sexuality struggle to identify appropriate services, and there is an absence of evidence-based interventions to promote sexual health and wellbeing in this population (45).

In transgender women after vaginoplasty, digital rectal examination will not necessarily allow examination of the prostate. In a study of 320 transgender women after undergoing vaginoplasty, digital examination of the prostate was only possible vaginally in 48% (46). Also, PSA levels in transgender women on GAH must be interpreted with caution and proper consideration must be given to their hormone regimen, testosterone levels, and whether they have undergone GAS. There are no studies on how to interpret PSA density or multiparametric MRI in patients on GAH, an important resource used to stratify risk of prostate cancer in cisgender males (39).

Besides the lack of screening guidelines, the etiology of prostate cancer in transgender women raises some questions. It is unclear how prostate cancer develops in androgen-deprived conditions in these patients. Six out of 11 case reports in the literature presented metastatic disease. It is thought that androgen receptor-mediated mechanisms or tumor-promoting effects of estrogen may be responsible (31). Reasons for the development of prostate cancer in transgender women have been hypothesized to include existing cancerous lesions prior to initiation of estrogen therapy, estrogen sensitive lesions, and androgen receptor variants (47). The long-term effects of GAH pose a potential challenge unique to transgender patients. GAH for transgender people is different from hormone replacement therapy for cisgender people in two ways: (I) when GAH is provided before surgical removal of the birth-sex gonads, the patient may have elevated serum levels of both masculinizing and feminizing hormones; (II) the effective dose of GAH can vary widely by individual patient, such that some have significantly higher serum levels of a particular hormone or its metabolites, which can increase (or decrease) risk of sex hormone-sensitive cancers (33). Another relevant aspect is that prolonged use of cross-sex hormones has been shown to have possible negative effects on ovarian and testicular function, so urologists should engage their transgender patients in discussion regarding their plans for future childbearing (12).

Prostate cancer with hormone therapy effect may not only be histologically subtle and thus be overlooked if not suspected, but also should not be assigned a Gleason score because this score would substantially overstate the biological potential. Therefore, like cis-male patients who have received androgen deprivation therapy for prostate cancer, transgender patients on hormone therapy for gender affirmation may be at risk for both under-recognition and over-grading of prostate cancer, particularly if the pathologist is not aware of the clinical history (48).

The treatment of prostate cancer in transgender women also has gaps in knowledge to provide evidence-based guidance for clinical decision-making in the management of these patients. Early and locally advanced prostate cancer in these patients warrants an individualized and thoughtful approach with input from patients’ reconstructive surgeons. Both surgical and radiation treatment for prostate cancer in these patients can profoundly impact the patient’s quality of life (31). For transgender women who have already undergone gender affirming surgery, prostate cancer treatment may again be complex. Radical prostatectomy after neovagina formation may lead to fistulae (rectovaginal or urethro-vesico-neovaginal), and radiotherapy can cause neovaginal stenosis and increase the risk of dyspareunia (46).

The specific needs of transgender women reinforce the importance of this knowledge on the part of urologists, even for those with large experience. The LGBT+ peculiarities have a direct impact on treatment satisfaction. These important aspects allow doctors to clarify possible consequences of the chosen therapeutic modality for prostate cancer, whichever technique is used. The decision-making process should take into consideration gender identity and sexual orientation. Therefore, the preoperative information must be directed towards transwomen´s reality. The execution of the surgery/radiotherapy itself is closely associated with the specific care, and furthermore, the postoperative follow-up and the techniques for reestablishing sexual life must be customized considering the peculiarities of the patient’s sexual orientation and sexual practices (1).

Finally, urologists must keep in mind that we are able to dramatically alter the health trajectories of these people. In addition to inspiring new studies, the authors of this article (1) have helped to eliminate barriers, thus promoting equal care and encouraging patients to seek medical help.

Remember that patients belonging to the LGBT+ community do not need judgments, curiosity, opinions about right or wrong concerning sexuality, but certainly they deserve a welcoming attitude by all staff members. They deserve to be treated with the same kindness and respect as all other patients. They would be pleased to hear and read their social names. Most importantly, do not forget that they have searched for a urologist with comprehensive knowledge and experience, believing that the practitioner will take into consideration all the peculiarities inherent to their individual characteristics, including gender identity and sexual orientation.
